# Treatment of Obesity and Diabetes Using Oxytocin or Analogs in Patients and Mouse Models

**DOI:** 10.1371/journal.pone.0061477

**Published:** 2013-05-20

**Authors:** Hai Zhang, Chenguang Wu, Qiaofen Chen, Xiaoluo Chen, Zhigang Xu, Jing Wu, Dongsheng Cai

**Affiliations:** 1 Department of Molecular Pharmacology, Diabetes Research Center, Institute of Aging, Albert Einstein College of Medicine, Bronx, New York, United States of America; 2 Department of Medicine, Endocrine Division, the Affiliated People’s Hospital of Jiangsu University, Jiangsu Province, Zhenjiang, P. R. China; Broad Institute of Harvard and MIT, United States of America

## Abstract

Obesity is important for the development of type-2 diabetes as a result of obesity-induced insulin resistance accompanied by impaired compensation of insulin secretion from pancreatic beta cells. Here, based on a randomized pilot clinical trial, we report that intranasal oxytocin administration over an 8-week period led to effective reduction of obesity and reversal of related prediabetic changes in patients. Using mouse models, we further systematically evaluated whether oxytocin and its analogs yield therapeutic effects against prediabetic or diabetic disorders regardless of obesity. Our results showed that oxytocin and two analogs including [Ser4, Ile8]-oxytocin or [Asu1,6]-oxytocin worked in mice to reverse insulin resistance and glucose intolerance prior to reduction of obesity. In parallel, using streptozotocin-induced diabetic mouse model, we found that treatment with oxytocin or its analogs reduced the magnitude of glucose intolerance through improving insulin secretion. The anti-diabetic effects of oxytocin and its analogs in these animal models can be produced similarly whether central or peripheral administration was used. In conclusion, oxytocin and its analogs have multi-level effects in improving weight control, insulin sensitivity and insulin secretion, and bear potentials for being developed as therapeutic peptides for obesity and diabetes.

## Introduction

Developing peptides to treat obesity and/or diabetes is a relatively recent advance, but of importance, the clinical success of this concept has been evidenced by the usefulness of intestinal peptide glucagon-like peptide-1 (GLP-1) in controlling obesity as well as diabetes [1]. Along this line, dipeptidyl peptidase-4 (DPP-4) inhibitors were developed to stabilize endogenous GLP-1, an approach which is effective in treating obesity and diabetes [2]. Also notably, combinational release and actions of multiple peptides including GLP-1 may account for, at least partly, the profound effects of bariatric surgery in treating obesity and diabetes [3]. In addition, bariatric procedures can have immediate effects in lowering blood sugar levels in diabetes prior to evident changes of body weight [4], [5], and this obesity-independent hypoglycemic effect has been related to the action of GLP-1 in promoting insulin section [3], [6]. Taken together, these recent advances call for explorations into new peptides and in particular neuropeptides since they have important and broad-range actions in regulation of body weight, insulin sensitivity and insulin secretion. Oxytocin (OXT) is a nine-amino acid neuropeptide produced by hypothalamic OXT neurons and released locally in the brain or systemically via their axonal terminals in the posterior pituitary, and the systemic action of OXT is known to mediate reproductive activities of females including laboring and lactation [7]. Recently, neurobiological research has led to the discovery of OXT’s reproduction-unrelated neuropsychiatric roles which are important for social recognition, pair bonding, emotion, trust, love and care [8]–[13]. Interestingly, our recent research demonstrated that the intra-brain action of OXT can lead to reversal of obesity as well as related glucose and insulin disorders in mouse models [14], [15]. Subsequently, similar anti-obesity effects of OXT were reported to occur in rat models [16], [17]. It is particularly worth mentioning that although the metabolic benefits of OXT is significantly attributed to the central action of this peptide [14], our studies and others showed that peripheral OXT delivery can work to exert anti-obesity effects [15], [16], and we further revealed that the underlying mechanism is related to the mechanism that peripherally delivered OXT can promote the intra-brain release of OXT to induce the metabolic actions [15]. Herein, grounded in our recent findings, we continued to investigate whether OXT has effects to treat obesity and related metabolic abnormalities in humans, and further explored in rodent models whether OXT and its analogs have anti-diabetic effects that could be dissociable from obesity control. Our results uncovered that OXT and its analogs have multiple therapeutic effects including weight control, lipid lowering, insulin sensitization and insulin secretion, and thus have druggable potentials of being developed as a new class of small peptides for treating obesity as well as diabetes that is related or unrelated to obesity.

## Materials and Methods

### Clinical study

This was a pilot clinical study registered through Chinese Clinical Trial Register (ChiCTR-TRC-12002884), and conducted in accordance with the Declaration of Helsinki, Good Clinical Practice guidelines, and other relevant regulations by the People's Republic of China. Study protocol and informed consent form were approved by the Ethics Committee of the affiliated People’s Hospital of Jiangsu University. Written informed consent was given to each patient.

#### Patients

Patients were recruited by clinicians through clinics of the People’s Hospital of Jiangsu University (Table 1). Estimate of sample size was determined using standard method and biomedical information. We predicted that the effect rate in OXT treatment group was greater than 50%, and the effect rate in placebo group was less than 15%. The settings of 80% power and α = 0.05 were used, and ∼25% dropout was estimated. Inclusion criteria: patients with body mass index (BMI) equal to or greater than 28 kg/m2, ages from 20 to 60 years, and who were willing and able to comply with study protocol. Exclusion criteria: 1) diabetes mellitus; 2) history of rhinitis or chronic respiratory diseases; 3) OXT allergy; 4) coronary heart disease, myocardial infarction, arrhythmia, or hypertension (blood pressures over 160/90 mmHg); 5) liver or kidney diseases; 6) other conditions including hematopoietic dysfunction, tumors and autoimmune diseases; 7) mental disorders; 8) having received steroid treatments within 3 months prior to this study; 9) having received OXT treatment within 30 days prior to this study; 10) planning to receive other weight loss treatments concomitant to this study; 11) drug or alcohol abuse; 12) females with pregnancy or lactation.

**Table 1:** Demographic information.

	Placebo ( n = 11)	Oxytocin (n = 9)
**Age in years, mean (SD)**	41.3 (10.2)	29.3 (8.7)
**Male %**	36.4	44.4
**Female %**	63.6	55.6
**Height, mean (SD)**	165.5 (6.2)	164.2 (6.6)
**Body weight, mean (SD)**	81.9 (7.4)	96.1 (17.2)
**BMI, mean (SD)**	30 (1.2)	36 (5.2)
**Waist circumference, mean (SD)**	100 (6.5)	112 (11.3)
**Hip circumference, mean (SD)**	108 (5.5)	114 (9.0)

#### Study approach

**Figure 1:**
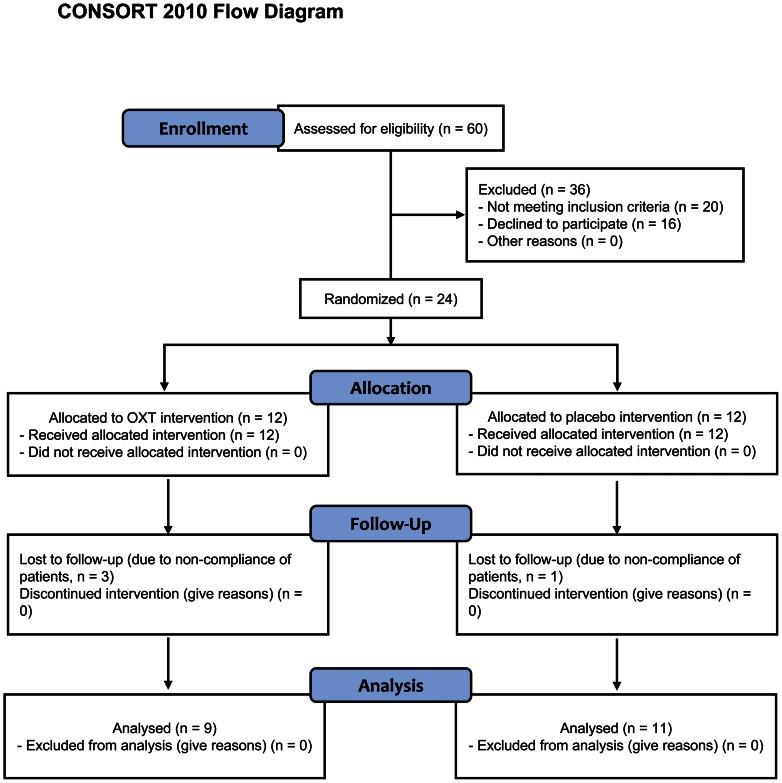
Study design and flowchart for clinical trial of intranasal OXT treatment. Patients with obesity that fit with the designed inclusion and exclusion criteria were recruited and randomly assigned into OXT vs. placebo treatment groups, and followed for body weight and related metabolic parameters during 8-week treatment duration.

Using randomized placebo-controlled design (Fig. 1), patients were recruited and randomly assigned into OXT vs. placebo (saline) treatment groups by clinicians/research assistants without being involved in study design or outcome/data analyses. Patients in OXT group received OXT nasal spray (Meike, Sichuan, China) at the dose of 24 units each time, 4 times a day (∼20 minutes before each of 3 meals and sleep, respectively) for a continuous 8-week period. Patients in placebo treatment group followed the same procedure using the identical spray device and labels. Patients were scheduled to have clinical visits at Week 4 and 8 to receive physical exams, blood and urine laboratory tests, measurements of body weight, waist/hip circumferences as well as blood glucose, insulin and lipids, and assessments of study implementation and adverse events (see additional information in Materials S1 and S2).

#### Statistical analysis

Statistical analyses were performed by investigators who did not have access to patients and were blind to the information during the entire course of the study. Body weight, BMI, and waist and hip circumferences, blood glucose and insulin levels, blood pressure, lipid profiles, and parameters of hepatic and renal functions were compared among different treatment points and between OXT and placebo groups. Repeated measures analysis was used to adjust dependence among repeated observations on the same patients. ANOVA and appropriate *post hoc* analyses were used for comparisons involving more than two groups, and two-tailed Student’s t tests were used for only 2-group comparisons. Clinical data were presented as mean ± SD. P<0.05 was considered significant.

#### Animal study

C57BL/6 mice were purchased from Jackson Laboratory. All mice were housed in standard conditions. High-fat diet was purchased from Research Diets, Inc. All animal procedures were approved by the Institutional Animal Care and Use Committee (IACUC) at the Albert Einstein College of Medicine. Mice were subjected to body weight measurement on a daily basis surrounding surgical procedures or drug treatments to monitor physical recovery or the effect of drug treatment on body weight.

#### Brain third-ventricle cannulation

Third ventricle cannulation procedure was previously described [18]. An ultra-precise small animal stereotactic apparatus (Kopf Instruments) was used to perform implantation of a guide cannula into the third ventricle of anesthetized mice, at the coordinates of 1.8 mm posterior to the bregma and 5.0 mm below the skull surface. Mice were allowed 1–2 weeks for post-operational recovery.

#### Drug treatment

OXT and [Ser4, Ile8]-OXT (Bachem Americas, Inc.) and [Asu1,6]-OXT (American Peptide Company, Inc.) were dissolved in the vehicle, aCSF. HFD-fed mice were twice injected with OXT or OXT analog via third-ventricle cannula (4 μg per injection) or i.p. (2 mg/kg body weight per injection), once at the beginning of overnight fasting and once in the morning prior to GTT. Injection of the vehicle was used as the control. For STZ model, adult C57BL/6 mice were daily injected with OXT or OXT analog vs. the vehicle via third-ventricle cannula (2 μg per injection) for 7 days, or i.p. (2 mg/kg body weight) vs. the vehicle for 3 days; all mice received daily i.p. injections of STZ (Sigma) at the dose of 40 mg/kg body weight for 4 days before these mice were subjected to GTT.

#### Blood tests

For glucose tolerance test (GTT), overnight-fasted mice received a single i.p. injection of glucose (2 g/kg body weight), and blood glucose levels were measured using OneTouch Ultra2 (LifeScan, Inc.). Fasting blood insulin levels were measured with Ultra Sensitive Mouse Insulin ELISA kits (Crystal Chem). Glucose-stimulated insulin secretion (GSIS) test was performed as previously described [19]. Overnight-fasted mice received a single intraperitoneal injection of glucose (3 g/kg body weight), and blood insulin levels were measured at 3 min and 20 min after glucose injection.

#### Statistical analysis

Two-tailed Student’s t tests were used for two-group comparisons. ANOVA and appropriate *post hoc* tests were used for comparisons that involved more than two groups. Data of animal studies were presented as mean ± SEM. P<0.05 was considered statistically significant.

## Results

### Treatment of obesity by OXT nasal spray in patients

**Figure 2:**
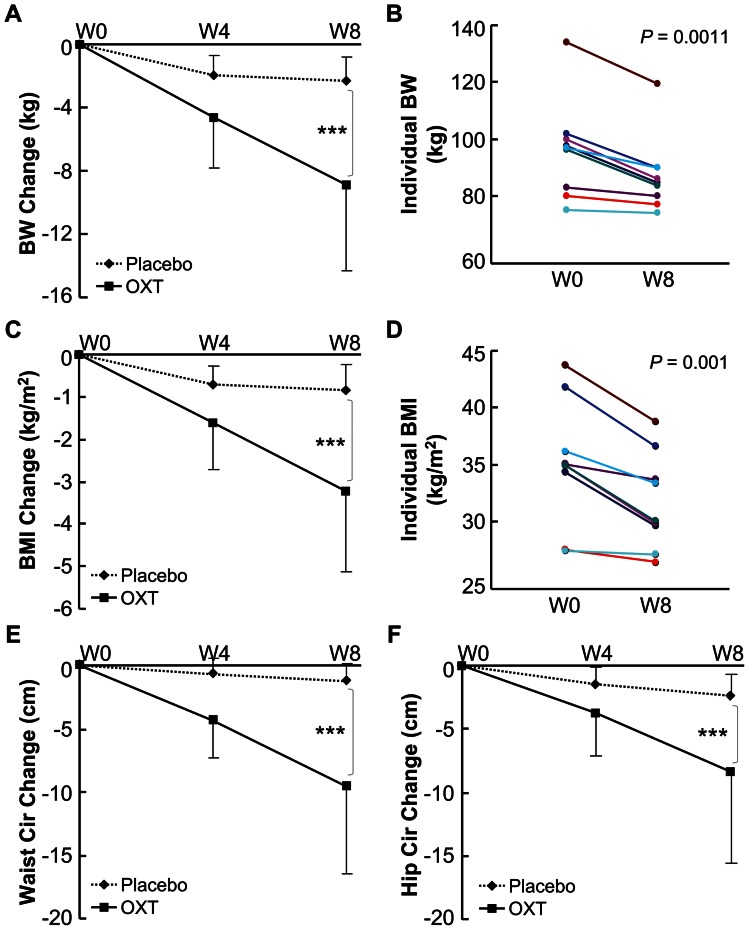
Effect of intranasal OXT treatment on obesity in humans. *A*–*F*: Patients with obesity received 8 weeks (W) of intranasal OXT vs. placebo treatment and were monitored for body weight (BW), BMI and waist and hip circumference (Cir) levels. Data show changes in BW (*A* and *B*), BMI (*C* and *D*), waist circumference (*E*), and hip circumference (*F*) between OXT and placebo treatment groups (*A, C, E* and *F*) or among individuals prior to (W0) and post 8-week (W8) OXT treatment (*B* and *D*). ***P<0.001; n = 9 for OXT treatment group, n = 11 for placebo treatment group. Error bars represent mean ± SD.

Using mouse models, our previous studies revealed that central administration of OXT can work to treat or prevent against dietary obesity [14], [15]. Of importance, our studies showed that peripheral administration of OXT can stimulate the central release of OXT to induce metabolic effects [15], provoking us to test if the method of peripheral OXT delivery could be developed to treat metabolic diseases in human patients. In this pilot clinical study, we employed intranasal spray of OXT vs. placebo (saline) to treat patients with obesity (MBI≥28 kg/m2) for 8 weeks, and treatment dose of OXT was 24 units each time, 4 times a day at ∼20 minutes before three meals and sleep. As demonstrated in Fig. 2A and 2B, we found that while placebo did not lead to a therapeutic effect against obesity, 4-week OXT treatment resulted in body weight reduction by 4.6±3.2 kg. This therapeutic effect continued to enlarge when the duration of OXT treatment increased to 8 weeks, showing that compared to the pre-treatment baseline levels, body weight of these patients dropped 8.9±5.4 kg (P<0.001). In general, the magnitude of weight loss induced by OXT treatment was more appreciable in patients with higher degree of obesity. BMI was calculated, and data showed 8-week OXT treatment reduced this parameter by 3.2±1.9 kg/m2 (P<0.001) (Fig. 2C and D). Consistently, weight loss by OXT treatment was evidently reflected by decreases in waist and hip circumferences of patients (Fig. 2E and F). In addition, despite that OXT is in clinical use for many decades, we assessed the safety of 8-week OXT treatment in our study. During the course of this study, no any adverse effects were reported from patients or found by our physical exams. Also as summarized in Table 2, OXT treatment did not cause any cardiovascular, liver or kidney dysfunctions, and if anything, hepatic function of patients was slightly improved, a possible outcome of obesity control which can reduce hepatic steatosis. Taken together, for the first time, we provided clinical evidence demonstrating that OXT nasal spray works to treat obesity in human patients. On the other hand, it should be pointed out that future studies of using larger samples and longer duration will help fully evaluate the efficacy and side effect profiles of OXT nasal spray in treating obesity and related diseases.

**Table 2:** Liver and kidney function.

	Placebo ( n = 11)			Oxytocin (n = 9)		
Treatment (weeks)	0	4	8	0	4	8
ALT (u/l), mean (SD)	28 (17)	29 (12)	28 (14)	36 (21)	34 (8.1)	30 (6.5)
AST (u/l), mean (SD)	27 (17)	25 (9.5)	24 (10)	26 (17)	23 (7.7)	20 (6.4)
Creatinin (umol/l), mean (SD)	75 (18)	71 (11)	71 (12)	76 (18)	76 (8.5)	75 (7.9)
Urea N (mmol/l), mean (SD)	6.0 (1.5)	5.8 (1.2)	5.7 (1.2)	5.2 (1.4)	5.0 (1.2)	4.9 (1.0)

### Reversal of obesity-related lipid disorders by OXT nasal spray in patients

**Figure 3:**
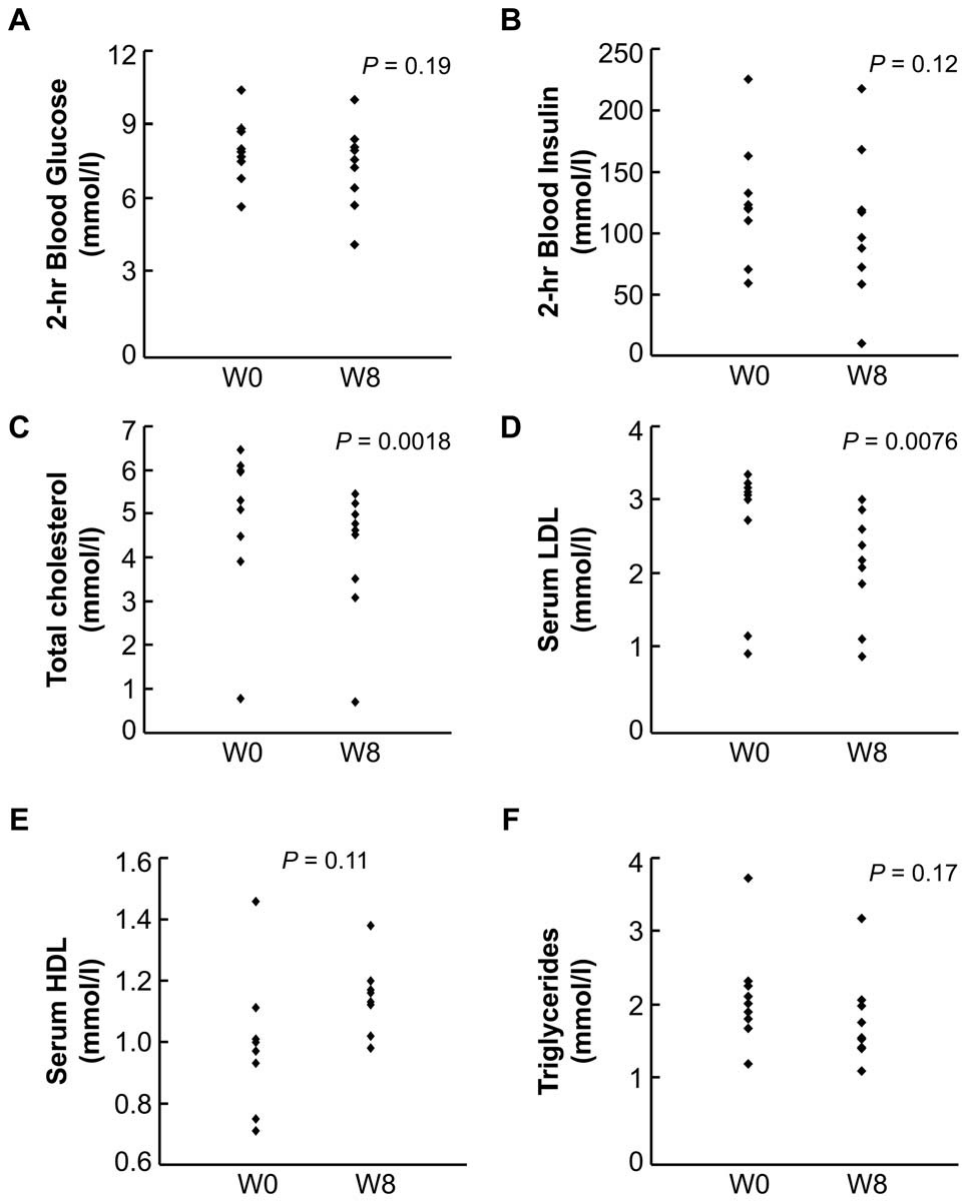
Effects of OXT treatment on obesity-related metabolic changes in humans. *A*–*F*: Patients with obesity received 8 weeks (W) of intranasal OXT vs. placebo treatment as described in Figure 1, and monitored for blood glucose, insulin and lipid profiles. Data show 2-hr postprandial blood glucose (*A*) and insulin (*B*), and fasting serum levels of total cholesterol (*C*), low-density lipoprotein (LDL) (D), high-density lipoprotein (HDL) (E) and triglycerides (TG) (F) of the individuals prior to (W0) and post 8-week (W8) OXT treatment. Statistical values for the differences between prior and post OXT treatment are provided in individual figures.

Clinical epidemiology has established that obesity in humans is associated with pre-type 2 diabetes (T2D) changes such as glucose intolerance, insulin resistance and hyperlipidemia. Thus, although this pilot clinical study did not involve patients with diabetes, we still monitored blood glucose, insulin and lipid levels of study subjects. We found that while OXT treatment did not affect fasting blood glucose or insulin levels, it tended to reduce postprandial glucose and insulin levels towards healthier profiles within the normal ranges (Fig. 3*A and B*). Interestingly, changes in postprandial glucose or insulin levels were not exactly correlated with reduction of body weight in these patients, perhaps suggesting that OXT could employ a body weight-independent mechanism to improve glucose and insulin homeostasis. Again, it should be noted that the design of this study excluded diabetic patients, and thus it remains to be tested if OXT nasal spray could treat diabetes in obesity-independent manner, in addition to the well-appreciated contribution of weight loss to diabetic intervention. In this clinical study, we also examined blood lipid profiles of patients, and interestingly found that OXT treatment significantly reduced serum LDL and cholesterol levels and tended to increase serum HDL levels (Fig. 3*C*–*F*). Altogether, our data indicated that OXT treatment can yield multiple benefits in weight balance and metabolic control in patients, and the approach of OXT nasal spray has a therapeutic potential to be used to treat obesity-associated lipid disorders in humans. In combination with data in Figure 2, our clinical observations lend a proof of principle to our hypothesis that OXT or its analogs can be developed to treat human obesity and related metabolic diseases.

### Reversal of insulin resistance in mice via central administration of OXT or analogs

**Figure 4:**
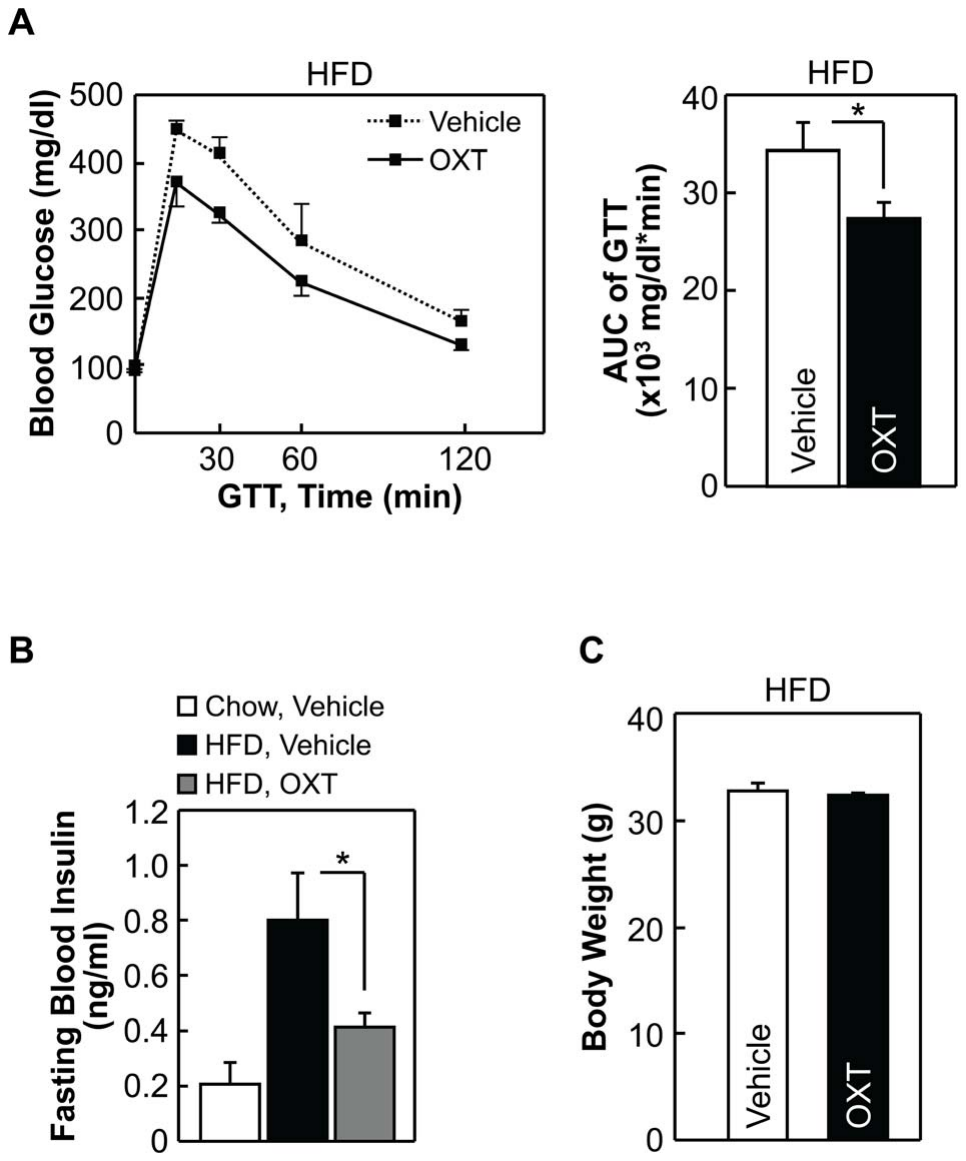
Acute central effects of OXT on obesity-related prediabetic changes in mice. *A*–*C*: C57BL/6 mice were kept on HFD feeding for 2 months to develop prediabetic disorders, and subsequently received third-ventricle injection of OXT or vehicle twice during an overnight fasting period. Data show the effects of drug treatment on glucose tolerance (*A*), fasting blood insulin levels (*B*) and body weight (*C*). *P<0.05; n = 5–8 (*A* and *C*) and n = 4–5 (*B*) mice per treatment group. Error bars represent mean ± SEM. GTT: glucose tolerance test, AUC: area under curve.

**Figure 5:**
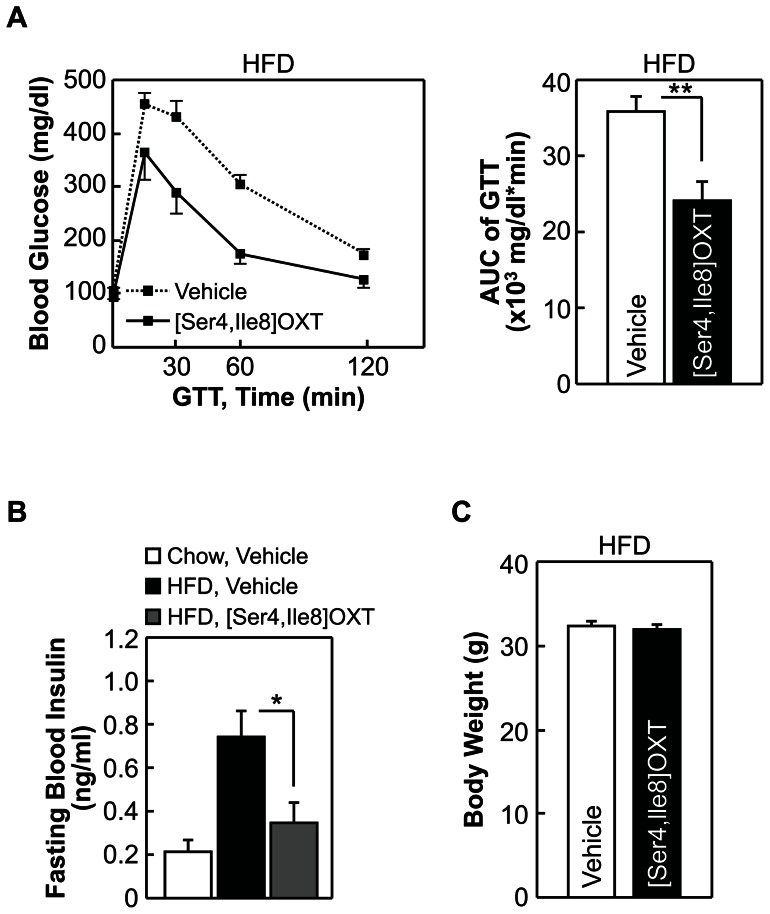
Acute central effects of [Ser4, Ile8]-OXT on obesity-related prediabetic disorders. *A*–*C*: C57BL/6 mice were maintained on HFD feeding for 2 months to develop prediabetic metabolic changes. After that, these mice received third-ventricle injection of [Ser4, Ile8]-OXT vs. vehicle twice during an overnight fasting period. Data demonstrate the effects of drug treatment on glucose tolerance (*A*), fasting blood insulin levels (*B*) and body weight (*C*). *P<0.05, **P<0.01; n = 5–8 (*A* and *C*) and n = 4–5 (*B*) mice per treatment group. Error bars represent mean ± SEM. GTT: glucose tolerance test, AUC: area under curve.

**Figure 6:**
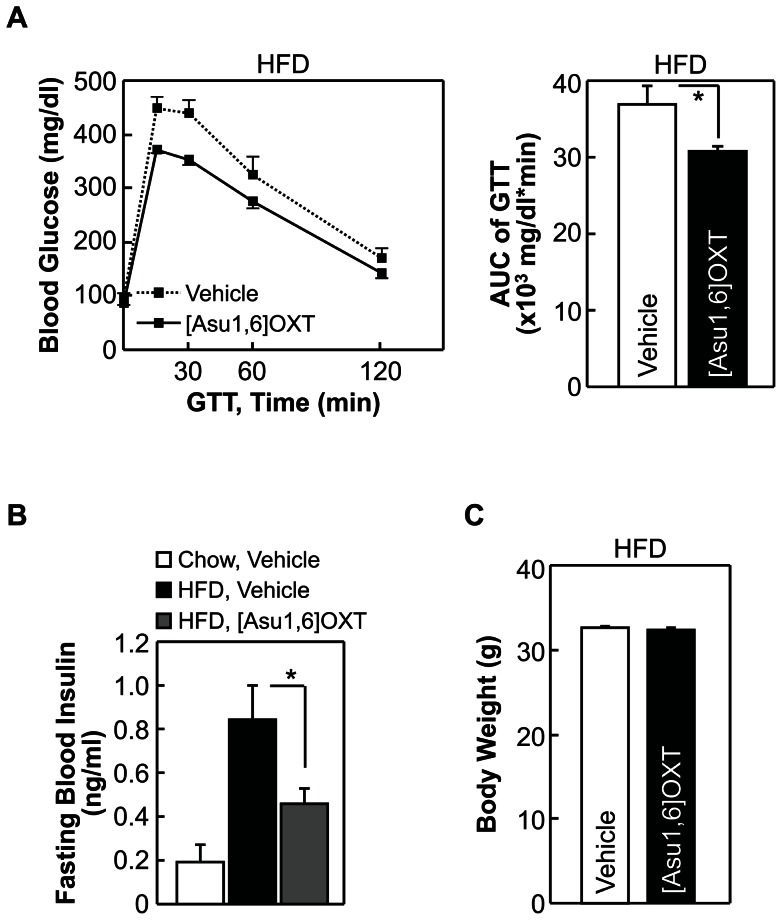
Acute central effects of [Asu1,6]-OXT on obesity-related prediabetic changes. *A*–*C*: C57BL/6 mice were fed on HFD feeding for 2 months to develop pre-diabetes, and then received third-ventricle injection of [Asu1,6]-OXT or vehicle twice during an overnight fasting period. Data show the effects of drug treatment on glucose tolerance (*A*), fasting blood insulin levels (*B*) and body weight (*C*). *P<0.05; n = 5–8 (*A* and *C*) and n = 4–5 (*B*) mice per therapy group. Error bars represent mean ± SEM. GTT: glucose tolerance test, AUC: area under curve.

Our previous study using mouse models had shown that enhancing the intra-brain action of OXT can prevent against the development of obesity and obesity-related diabetic symptoms such as insulin resistance, glucose intolerance, pancreatic islet hypertrophy and steatosis against dietary overnutrition [14]. In this study we further asked whether the potential effect of OXT in treating diabetes is completely dependent on obesity control by OXT. While it will take efforts to clinically test this idea, we evaluated this hypothesis through using acute experimental conditions for a pre-T2D mouse model induced with chronic high-fat diet (HFD) feeding. In the experiment, these mice received third-ventricle injections of OXT during an overnight fasting period before they were subjected to glucose tolerance test (GTT). We found that OXT treatment significantly improved glucose intolerance (Fig. 4*A*) and fasting blood insulin levels (Fig. 4*B*) of mice, and this effect was observed without involving body weight change (Fig. 4*C*) since the experimental duration was only an overnight period. Thus, central OXT treatment can employ a body weight-independent manner to reverse insulin resistance and glucose intolerance. We further tested if the effects of OXT could be recapitulated by OXT analogs. For this purpose, we examined [Ser4, Ile8]-OXT, since it contains glutamine to serine substitution at amino acid position 4 which is predicted to strengthen α-helical structure and stabilize peptide conformation [20] and thus increase drug duration. Using the pre-T2D model described above, we found that compared to native OXT, [Ser4, Ile8]-OXT yielded more pronounced effects in correcting glucose intolerance (Fig. 5*A*) and hyperinsulinemia (Fig. 5*B*). Thus, with the structural modifications, [Ser4, Ile8]-OXT can potentially outperform OXT in treating metabolic diseases. In addition, we comparatively examined [Asu1,6]-OXT, an OXT analog which was reported to have 70% activity of native OXT [21]. Using the same prediabetic mouse model, we consistently found that [Asu1,6]-OXT treatment improved glucose intolerance (Fig. 6*A*) and lowered fasting blood insulin levels (Fig. 6*B*), and both effects were independent of body weight (Fig. 6*C*). The therapeutic effects of [Asu1,6]-OXT seemed to be comparable with the effects of OXT (Fig. 4), but weaker than the effects of [Ser4, Ile8]-OXT (Fig. 5). Altogether, these results indicated that OXT peptide engineering can help optimize the therapeutic effects for metabolic diseases.

### Improvement of insulin secretion in mice via central administration of OXT or analogs

**Figure 7:**
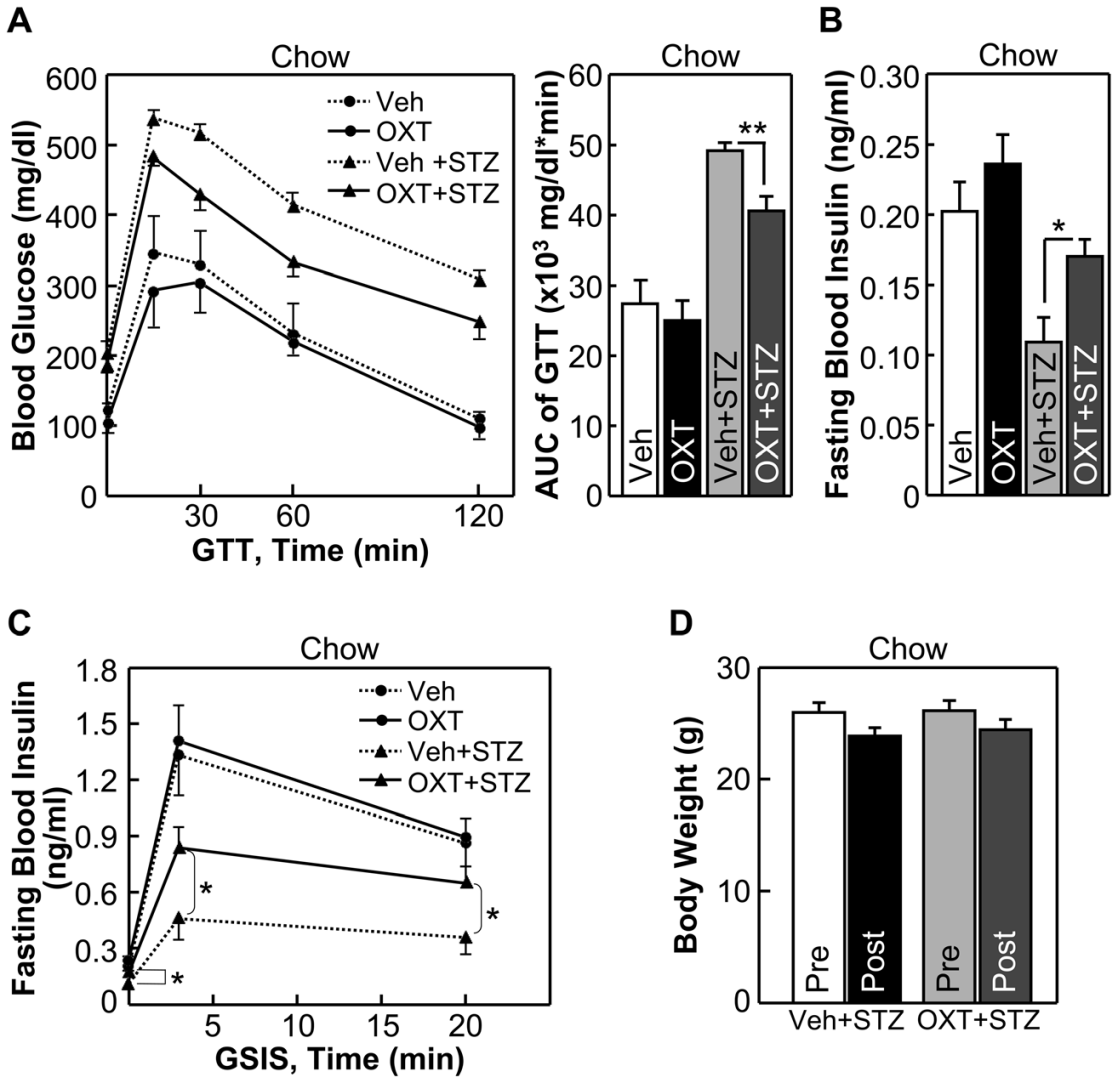
Central effects of OXT on glucose and insulin in STZ-induced diabetic mice. *A*–*D*: C57BL/6 mice under normal chow feeding received 7-day third-ventricle delivery of OXT vs. vehicle, and were subjected to daily i.p. injections of STZ from Day 4 to 7 to impair insulin secretion. Sub-groups of OXT vs. vehicle-treated mice that did not receive STZ injections were included to provide baseline levels. Data show the effects of OXT treatment on glucose tolerance (A), fasting blood insulin levels (*B*), first-phase and second-phase glucose-stimulated insulin release (GSIS) (*C*) and body weight (*D*). *P<0.05, **P<0.01; n = 6 mice per group (Veh + STZ vs. OXT + STZ), and n = 4 mice per group (Veh vs. OXT). Error bars represent mean ± SEM. GTT: glucose tolerance test, AUC: area under curve.

**Figure 8:**
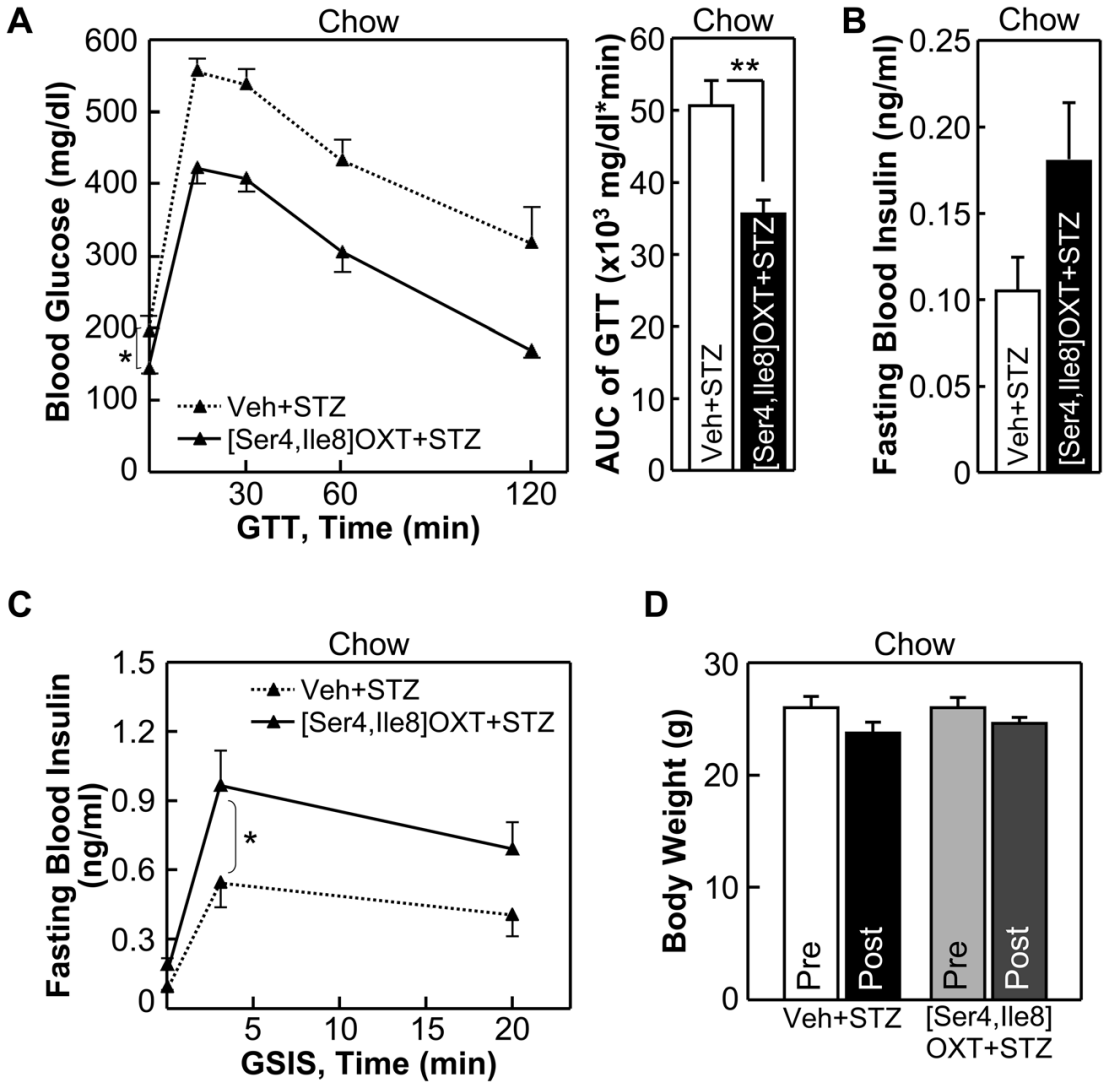
Effects of [Ser4, Ile8]-OXT on glucose and insulin in STZ-induced diabetes. *A*–*D*: Chow-fed C57BL/6 mice received 7-day third-ventricle administration of [Ser4, Ile8]-OXT vs. vehicle, and were subjected to daily i.p. injections of STZ from Day 4 to 7 to impair insulin secretion. Data show the effects of OXT treatment on glucose tolerance (A), fasting blood insulin levels (*B*), first-phase and second-phase glucose-stimulated insulin release (GSIS) (*C*) and body weight (*D*). *P<0.05, **P<0.01; n = 6 mice per group. Error bars represent mean ± SEM. GTT: glucose tolerance test, AUC: area under curve.

**Figure 9:**
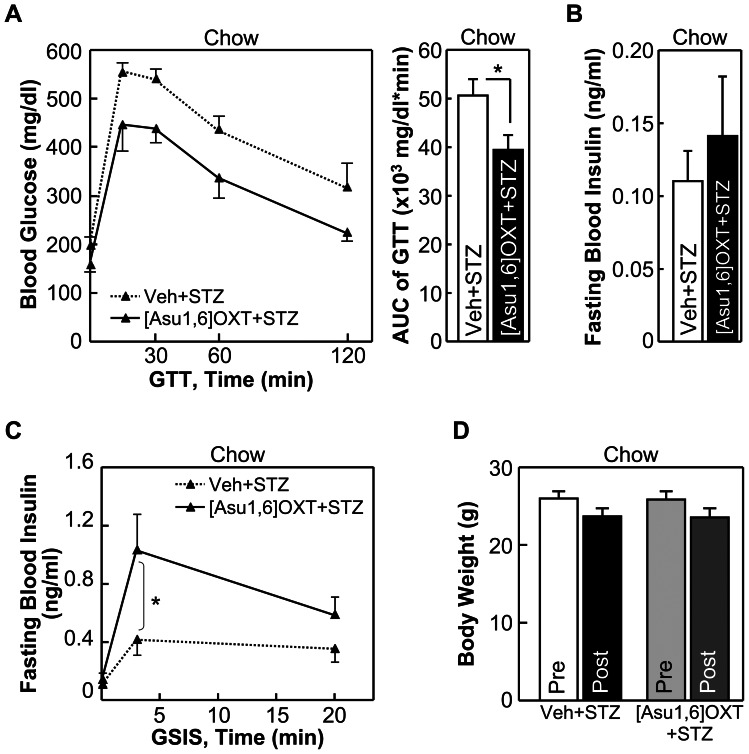
Effects of [Asu1,6]-OXT on glucose and insulin in STZ-induced diabetes. *A*–*D*: C57BL/6 mice fed on a normal chow received 7-day third-ventricle injection of [Asu1,6]-OXT vs. vehicle, and were subjected to daily i.p. injections of STZ from Day 4 to 7 to impair insulin secretion. Data show the effects of OXT treatment on glucose tolerance (*A*), fasting blood insulin levels (*B*), first-phase and second-phase glucose-stimulated insulin release (GSIS) (*C*) and body weight (*D*). *P<0.05; n = 6 mice per treatment group. Error bars represent mean ± SEM. GTT: glucose tolerance test, AUC: area under curve.

Next we studied if OXT and its peptide analogs could provide a protective effect against impaired insulin secretion which is known to underlie the development and progression of both T2D and type-1 diabetes (T1D). Our recent work showed that mice with genetically enhanced OXT function were significantly protected from overnutrition-induced prediabetic damages of pancreatic beta cells [14], although in that study we did not address the question of whether such a protection for beta cells was a primary effect of OXT or a secondary effect of obesity control. Herein, to study if OXT treatment has a beneficial effect on beta cells, we used streptozotocin (STZ)-induced diabetic mouse model which suffers from beta cell damage and insulin secretion impairment which resemble T1D and late-stage T2D, but does not have overeating or obesity. In this study, chow-fed C57BL/6 mice were injected with OXT via the third ventricle consecutively for 7 days, and from Day 4 to Day 7, these mice received daily i.p. injections of STZ to induce moderate beta cell damages., Results showed that as a result of STZ-impaired insulin secretion, vehicle-treated mice displayed hyperglycemia and glucose intolerance; however, OXT treatment led to significant improvements in glucose tolerance (Fig. 7*A*) and blood insulin levels (Fig. 7*B*). Using glucose-stimulated insulin secretion (GSIS) test, we found that OXT treatment partially prevented STZ from impairing first-phase and second-phase insulin secretion (Fig. 7*C*). Of note, because this study was based on normal chow feeding, and OXT treatment did not significantly affect body weight of mice (Fig. 7*D*), the action of OXT in promoting insulin secretion in these animals was not attributed to weight control. Next, we tested whether OXT analogs might act similarly to promote insulin secretion and thus maintain systemic glucose homeostasis. Using the same STZ-induced diabetic model and the same drug treatment paradigm, we found that [Ser4, Ile8]-OXT treatment significantly prevented STZ-induced glucose intolerance (Fig. 8*A*), and this effect was accounted for by the improvement on insulin secretion (Fig. 8*B and C*) rather than body weight (Fig. 8*D*). [Asu1,6]-OXT also appreciably, though to a lesser degree, improved glucose tolerance (Fig. 9*A*) and insulin secretion (Fig. 9*B and C*) without involving body weight changes (Fig. 9*D*). To summarize, these observations indicate that OXT and OXT analogs have potentials to improve insulin secretion to counteract glucose disorders in diabetes.

### Anti-diabetic treatment in mice through peripheral injection of OXT

**Figure 10:**
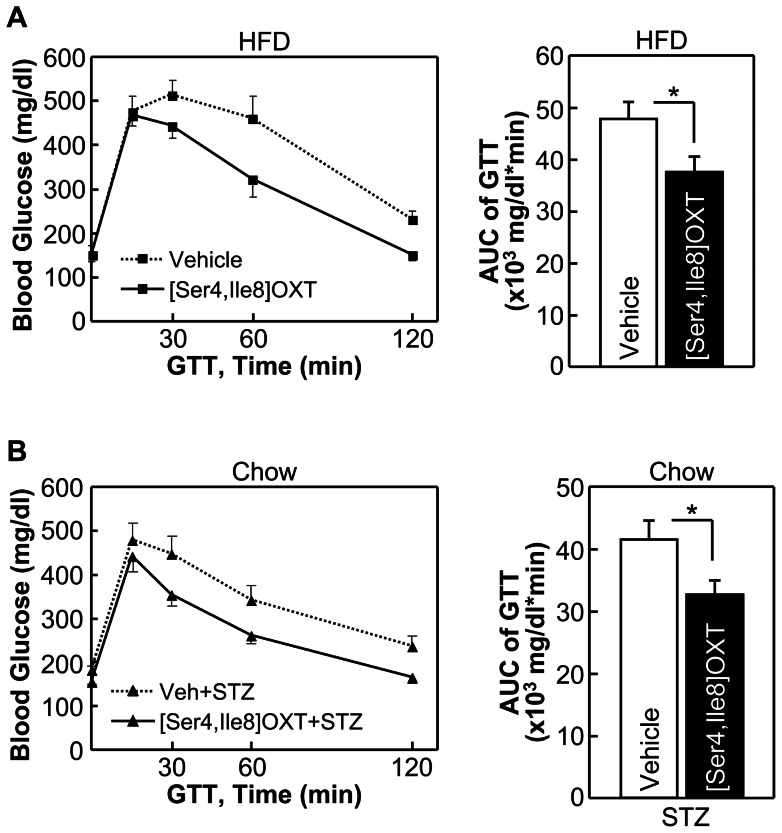
Effects of peripheral [Ser4, Ile8]-OXT delivery in treating diabetic changes. *A*: Adult C57BL/6 mice were maintained on HFD feeding for 2 months, and then received daily i.p. injections of [Ser4, Ile8]-OXT or vehicle. Data show the effects of drug treatment on glucose tolerance. *B*: C57BL/6 mice under normal chow feeding received daily i.p. injections of [Ser4, Ile8]-OXT vs. vehicle and injections of STZ as described in the method. Data show the effects of [Ser4, Ile8]-OXT treatment on glucose tolerance. *P<0.05; n = 6 mice per treatment group. Error bars represent mean ± SEM. GTT: glucose tolerance test, AUC: area under curve.

The above studies suggested that in addition to obesity control, OXT and its analogs can work in the brain to independently improve systemic insulin sensitivity and insulin secretion and thereof counteract against diabetes. However, given that drug delivery via brain injection is impractical in humans, we further evaluated whether peripheral OXT delivery could be workable. The rationale for this possibility includes that, the blood-brain barrier (BBB) does not completely block the passage of OXT [22]-[27], and more importantly, OXT when peripherally administrated can lead to activation of hypothalamic OXT neurons to induce central OXT release [15]. Indeed, our clinical data in Figures 2 and 3 already showed that OXT can be administrated peripherally to reduce obesity and related lipid abnormalities. In this background, we performed experiments to test whether the central actions of OXT in improving glucose and insulin homeostasis could be achieved through the approach of peripheral drug delivery. Our test focused on [Ser4, Ile8]-OXT, since it seemed to yield the strongest therapeutic effects as demonstrated in Figs. 4 to 9. First, using HFD feeding-induced prediabetic model, we found that [Ser4, Ile8]-OXT treatment over an overnight period led to improvement of glucose tolerance (Fig. 10*A*). In parallel, STZ-induced diabetic model was tested, and to do so, chow-fed C57BL/6 mice were subjected to i.p. injections of [Ser4, Ile8]-OXT and injections of STZ as described in the method. Data revealed that [Ser4, Ile8]-OXT treatment reduced the magnitude of STZ-induced glucose intolerance (Fig. 10*B*). Thus, OXT analogs can be delivered peripherally to improve glucose and insulin profiles, and as suggested by the success of reducing human obesity by peripheral OXT delivery (Figs. 2 and 3), our findings presented in this work propose OXT analogs as a class of peptide targets for the therapy of metabolic diseases including obesity and its related or unrelated diabetes.

## Discussion

### Multi-level therapeutic benefits of OXT and analogs in treating obesity and diabetes

OXT is a small neuropeptide produced by OXT neurons and released locally in the brain and systemically into the circulation. While the systemic action of OXT is important for female reproductive activities [7], recent neurobiological research revealed that brain actions of OXT are crucial for a battery of neuropsychiatric activities in both genders such as sociality, pair bonding, emotion, trust, love and care [8]-[13]. Our research further demonstrated that OXT can work in the brain to improve the neural regulation of metabolism and thereby counteract obesity and obesity-related metabolic abnormalities in mouse models [14], [15]. These findings provoked us to further assess whether the metabolic effects of OXT observed in animals could be reproduced in human patients. Herein, we performed a pilot clinical study focusing on patients with obesity, and our results showed that OXT nasal spray effectively reversed obesity as well as obesity-related lipid disorders. In addition to these metabolic effects, we noted that OXT treatment tended to improve blood glucose and insulin homeostasis; under this scope, while clinical studies based on diabetic patients are needed, we employed mouse models to examine if OXT could use treat diabetes. To dissociate the possible anti-diabetic action of OXT from its weight loss-inducing effect, we used acute experimental paradigm for mouse models of HFD-induced prediabetes or STZ-induced diabetes. Our data proved that OXT and its analogs have the actions to reverse insulin resistance and improve insulin secretion in these mouse models. As known, T2D develops when insulin secretion is impaired to a level that cannot compensate for systemic insulin resistance, and the presence of obesity can potentiate the progression of both insulin resistance and impaired insulin secretion. Thus, the collection of our data in this work, together with our recent findings [14], [15], demonstrated that the benefits of OXT in treating metabolic diseases are multi-faceted, range from obesity control to body weight-independent improvements of insulin sensitivity and insulin secretion. Admittedly, our experiments in this work did not address the underlying signaling mechanisms, but we speculate that multiple regulatory pathways could be involved. For example, since OXT neurons express melanocortin receptor-4 and respond to feeding-regulatory melanocortin signals [28]-[30], the brain action of OXT can contribute to improvement of these pathways in regulation of feeding and energy balance. Regarding how OXT works in the brain to control peripheral insulin sensitivity and insulin secretion, given the recently appreciated roles of central-peripheral axes for the metabolic functions of various peripheral tissues [31], [32], OXT in the brain may employ the autonomic nervous system and neuroendocrine pathways to impact on peripheral tissues such as the liver and pancreatic islets to improve insulin sensitivity and insulin secretion in these places.

### Effectiveness of peripherally delivering OXT or analog for treating obesity and diabetes

Peptides, especially those that naturally exist in bio-systems, are increasingly recognized for therapeutic potentials to treat human diseases in terms of effectiveness and safety; however, developing peptidyl drugs to target the central nervous system is still a pharmaceutical challenge. Based on current technology, direct intra-brain delivery is impractical for human beings, whereas peripherally delivered peptides often cannot effectively reach the central sites due to the physical and biological blockade of the BBB. As of now, successful cases of the central nervous system-targeting drugs are a few low-molecular weight (∼0.3KD) molecules that treat mental disorders or sleep disorders. OXT is a naturally existing peptide in mammals and has shown central anti-obesity and anti-diabetic effects per our rodent studies. Here, following our recent work showing the effect of peripheral OXT delivery in treating obesity in mice, the current work demonstrated that OXT nasal spray can effectively treat human obesity and the related lipid disorders. Along this line, our animal studies further revealed that peripheral administration of OXT analog can use a body weight-independent manner to improve both insulin sensitivity and insulin secretion in two diabetic/pre-diabetic models. Thus, with the recognition that intra-brain action of OXT is important for metabolic intervention, it is highly clinically relevant to see that OXT or its analogs can be delivered via peripheral administration to exert similar effects. We think the mechanism for the effects of peripheral OXT delivery is at least related to the central-peripheral crosstalk mediated by OXT, for instance, in addition to a possible fractural penetration of OXT across the BBB [22]-[27], we recently demonstrated that OXT when peripherally administrated can lead to activation of hypothalamic OXT neurons to induce central OXT release [15]. Based on findings in our studies, next step pharmaceutical and clinical studies are warranted to design OXT analogs that have optimal therapeutic effects via peripheral administration to treat obesity, diabetes and related metabolic diseases.

### Potential off-target benefits of OXT and OXT analogs in treating obesity and diabetes

The therapeutic advantages of OXT and its analogs in treating obesity and diabetes can be further increased given that OXT has social-neuropsychiatric benefits which can possibly aid to control metabolic disease. Though initially only recognized for functions in inducing female uterine contractions and milk let-down, OXT has recently been found to be important for social recognition, pair bonding, love and care in mammals and for mental and emotional well-being in humans [8]-[13]. In line with these understandings, OXT is currently under clinical trials for diseases such as autism, anxiety disorder, depression, drug abuse, and schizophrenia [33], [34]. These advances in clinical studies could be meaningful for our goal of developing OXT to treat obesity and diabetes, because there are strong correlations between metabolic diseases (such as obesity and diabetes) and mental disorders such as schizophrenia, bipolar disorders and severe depression [35]. Indeed, mental dysfunctions represent a type of risk factors for the development of obesity and diabetes, which probably involves certain brain alterations of metabolic control in addition to the issues such as poor lifestyle and nutrition management [36]-[38]. It is particularly worth mentioning that OXT has a therapeutic advantage over antipsychotic drugs in that OXT treatment decreases rather than increases food intake [14]. Hence, the improvement of mental and social-behavioral conditions by OXT could additionally facilitate the control of obesity and diabetes in the patients, and these benefits can further increase the attractiveness of developing OXT analogs to treat obesity and diabetes perhaps from neuropsychiatric point of mechanism.

## Supporting Information

Material S1 **Study protocol for using OXT nasal spray to treat human obesity.**
(PDF)

Material S2 **Consort checklist for using OXT nasal spray to treat human obesity.**
(PDF)
