# Supplementary material for: Treatment of Obesity and Diabetes Using Oxytocin or Analogs in Patients and Mouse Models
Source: PLoS One. 2013 May 20;8(5):e61477. doi: 10.1371/journal.pone.0061477 (PMC3658979; doi:10.1371/journal.pone.0061477)
Supplement: Material S1 — Study protocol for using OXT nasal spray to treat human obesity. (PDF) [file pone.0061477.s001.pdf]

Study the use of oxytocin nasal spray in treating human obesity

1. Study background and rationale

With the development of social economy and people lifestyle changes, morbidities of obesity and related diabetes are rising and widely recognized as one of the most challenging contemporary threats to public health; however, there is still lack of ideal treatment means so far. Therefore, it is important significance to explore new drug to treat obesity. Oxytocin is a bioactivative substance secreted by posterior pituitary gland. It can assist parturition and stop postpartum bleeding. Research shows that oxytocin not only causes uterine contraction, but also has a variety of biological effects. For example, oxytocin can improve children autism, and has antidepressant effect. Recent studies showed that oxytocin can reduce weight of obese animals, and can improve blood glucose, but whether oxytocin has these metabolic effects in the human is still not clear. Therefore, the current study will investigate if oxytocin can treat human obesity. This study can potentially significantly improve clinical treatment for obesity and related diabetes. Many historical weight-loss drugs have been abandoned because of serious toxic effects. For example, aminorex causes pulmonary hypertension, fenfluramine and dexfenfluramine causes valvulopathy, phenylpropanolamine causes stroke, rimonabant causes suicidal behavior, and most recently sibutramine is found to cause myocardial infarction and stroke. Oxytocin has been an affordable drug in human clinical use for many years, and there are not any side effects observed for heart, brain, lung, liver, kidney and other major organs. Therefore, if oxytocin is proven to treat human obesity, it will benefit the majority of obese patients, and create great social and economic benefits.

2. Research Objective

The aim of current study is to investigate the effect and safety of oxytocin for treating obesity.

3. Research Design

Patients with obesity in accordance with the inclusion and exclusion criteria are recruited and divided into placebo vs. oxytocin treatment. Randomized cased control study design is used.

4. Inclusion Criteria

1) Body mass index (BMI, body weight in kilograms divided by the square of height in meters) equal to or greater than 28 kg/m^2;
2) Ages between 20 to 60 years (1:1 male to female ratio);
3) All patients provide written informed consent prior to participating in the study;
4) Being willing and able to comply with the study protocol.

5. Exclusion Criteria

1) Diabetes mellitus; 2) History of rhinitis or chronic respiratory diseases; 3) Allergic to test drug; 4) With conditions of coronary heart disease, myocardial infarction, severe arrhythmia, liver, kidney or hematopoietic system dysfunctions (for example, systolic/diastolic blood pressures over 160/90 mmHg; ALT 2-fold higher than the upper limit of normal range; BUN and Cr values above the normal range), or severe mental disorders; 5) Tumors; 6) Auto-immune diseases; 7) Receiving systemic steroid treatments within 3 months immediately

before this study; 8) Receiving oxytocin treatment within 30 days immediately before this study; 9) Females being pregnant or lactating; 10) Drug and/or alcohol abuse; 11) Having or planning to receive other weight loss treatments, including medication, diet or exercise; 12) Unwilling to follow study arrangements or quitting from the study.

6. Randomization and blindness

Patients are recruited at clinics of the Affiliated People's Hospital of Jiangsu University and randomly assigned into OXT vs. placebo treatment group using simple randomization by clinicians/research assistants without being involved in research design or outcome/data analyses. The randomization and grouping information are kept blind to researchers who design the research and who perform outcome/data analyses until the study is completed. Drug vs. placebo are packed in the identical delivery spray device and labels, and are distributed to patients, and thus patients are blind to intervention information. At the end of study, results are statistically analyzed by researchers who have no patient contact and are blind to all related information during the entire course of the clinical study.

7. Intervention

Patients are randomly assigned to oxytocin treatment group vs. placebo group, and receive intranasal spray of oxytocin (24 units) vs. placebo (saline) at ~20 minutes before breakfast, lunch, dinner and night sleep, thus 4 times treatment totally per day.

8. Study Procedure

This study includes a 2-week screening period followed by 8-week treatment period. Patients that are recruited to the study are asked to come back to the clinic at Week 4 and 8 following the beginning of the intervention.

Visit 1 (Screening, 2 weeks prior to treatment):

- Collect and analyze medical history, perform physical examination, preliminarily assess patients for inclusion criteria. If patient preliminarily is in accordance with inclusion criteria, ask him/her to read the informed consent and sign it;
- Examine exclusion criteria;
- Document information including vital signs, height, weight, waist and hip circumferences;
- Collect blood sample before and after standard meal (100 g instant noodles), and test fasting glucose, 2-h postprandial blood glucose levels, lipid profile, hepatorenal function, fasting and 2-h postprandial plasma insulin, female patients need pregnancy test. All patients take electrocardiogram;
- Provide study diary and treatment guidance;
- Inform patients bring the diary to the research center on next visit.

Visit 2 (Week 0)

- Check the inclusion and exclusion criteria;
- Document patient information including vital signs, weight, waist and hip circumferences;

- Examine patient diaries, record adverse events;
- Patients that are in accordance inclusion and exclusion criteria are subjected to randomized allocation into OXT vs. placebo groups;
- Guide and provide new diaries;
- Tell patients to bring drug bottles and diaries back to research center when they visit next time.

Visit 3 (Week 4)

- Collect patient information, physical examination, measure weight, waist and hip circumferences;
- Collect blood samples before and after a standard meal, measure fasting and 2-h postprandial blood glucose levels, examine liver and kidney functions;
- Record adverse events;
- Guide and provide new diaries;
- Check patient compliance to study protocol, distribute new drug bottles;
- Tell patients to bring drug bottles and diaries back to research center when they visit next time.

Visit 4 (Week 8)

- Collect patient information, physical examination, measure weight, waist and hip circumferences;
- Collect blood samples before and after a standard meal, measure fasting and 2-h postprandial blood glucose levels, lipid profile, hepatorenal functions, fasting and 2-h postprandial plasma insulin levels;
- Record adverse events;
- Check patient compliance to study protocol.

Follow-up

Patients will continue to be followed up for up to 6 months, and assessments include physical examination, blood and urinary laboratory tests, measurements of body weight, waist and hip circumferences, blood glucose and lipids, liver and kidney functions, and inquires of any post-trial adverse events.

9. Endpoints and analysis

Statistical analyses are performed by investigator(s) who are blind to the information during the course of clinical study. Primary endpoints are reduction of body weight, BMI, and waist and hip circumferences, and secondary readouts include blood glucose, insulin and lipid profiles. Power of 80%, α value of 0.05, effect of greater than 50%, placebo effect of lower than 15%, and ~20% drop rate are used to calculate sample size. Repeated measures analysis is used to adjust dependence among repeated observations on the same patients. ANOVA and appropriate *post hoc* analyses are used for comparisons involving more than two groups, and two-tailed Student's t tests are used for only 2-group comparisons. Data are presented as mean ± SD. $P < 0.05$ is considered statistically significant.
